# Supplementary material for: Monitoring body condition score of reintroduced banteng (Bos javanicus D’Alton, 1923) into Salakphra Wildlife Sanctuary, Thailand
Source: PeerJ. 2020 Apr 23;8:e9041. doi: 10.7717/peerj.9041 (PMC7183756; doi:10.7717/peerj.9041)
Supplement: Supplemental Information 2 — Table S1 The scoring criteria for seven body compositions of the banteng (Bos javanicus) in Salakphra Wildlife Sanctuary. [file peerj-08-9041-s002.docx]

*Supplement Table 1* The scoring criteria for seven body compositions of the banteng (*Bos javanicus*) in Salakphra Wildlife Sanctuary.

| **Body composition** | **Body condition scoring system (BCS)*** | | | | |
| --- | --- | --- | --- | --- | --- |
|  | **1** | **2** | **3** | **4** | **5** |
| Neck | Very flat | Starting to round | Slightly rounded | Rounded | Full and rounded |
| Dewlap | Non-existent | Very small | Present | Fat apparent | Large flap of skin |
| Shoulders covered with soft tissue | Bones are prominent | In some part | Almost | Well | Very well |
| Vertebrae covered with soft tissue | Prominent all along backbone | Easily seen | Visible with some | Shadows can be seen | Not visible |
| Ribs covered by some tissue | Very prominent | Easily seen | Can be seen | Rarely visible | Not visible |
| Hindquarters | Flat at top, dished at pins and hooks | Flat at top with obvious hooks and pins | Slightly rounded, hooks still visible | Rounded, hooks are not visible | Rounded at top, flushed with tissue at hooks |
| Tail head | Very flat | Flat | Slightly rounded | Rounded | Rounded with small mounds of soft tissue |
