# Supplementary material for: Monitoring body condition score of reintroduced banteng (Bos javanicus D’Alton, 1923) into Salakphra Wildlife Sanctuary, Thailand
Source: PeerJ. 2020 Apr 23;8:e9041. doi: 10.7717/peerj.9041 (PMC7183756; doi:10.7717/peerj.9041)
Supplement: Supplemental Information 3 — Table S2 The seven body compositions of the banteng (Bos javanicus) in each individual, sex, age, year of reintroduction in Salakphra Wildlife Sanctuary. [file peerj-08-9041-s003.docx]

*Supplement Table 2* The seven body compositions of the banteng (*Bos javanicus*) in each individual, sex, age, year of reintroduction in Salakphra Wildlife Sanctuary.

| **ID** | **Sex** | **Age** | **year** | **Season** | **Neck** | **Dewlap** | **Shoulder** | **Vertebrae** | **Rib** | **Hindquarter** | **Tail head** | **Mean** |
| --- | --- | --- | --- | --- | --- | --- | --- | --- | --- | --- | --- | --- |
| PM | Male | 7 | 1 | Wet | 3 | 2 | 3 | 4 | 3 | 2 | - | 2.8±0.8 |
| PM | Male | 7 | 1 | Wet | 2 | 2 | 3 | 3 | 3 | 2 | 3 | 2.6±0.5 |
| PM | Male | 7 | 1 | Wet | 2 | 2 | 3 | 3 | 3 | 2 | - | 2.5±0.5 |
| PM | Male | 7 | 1 | Wet | 3 | 2 | 3 | 4 | 3 | 2 | 4 | 3±0.8 |
| PM | Male | 7 | 1 | Wet | 3 | 2 | 3 | 4 | 3 | 2 | 4 | 3±0.8 |
| KO | Male | 5 | 1 | Dry | 4 | 3 | 4 | 4 | 3 | 3 | 3 | 3.4±0.5 |
| KO | Male | 5 | 1 | Wet | 3 | 3 | 3 | 4 | 4 | 3 | 3 | 3.3±0.5 |
| PU | Male | 5 | 1 | Wet | 2 | 2 | 3 | 3 | 2 | 3 | 3 | 2.6±0.5 |
| PU | Male | 5 | 1 | Wet | 3 | 3 | 3 | 4 | 4 | 3 | 3 | 3.3±0.5 |
| PU | Male | 5 | 1 | Dry | 3 | 4 | 4 | 3 | 3 | 3 | - | 3.3±0.5 |
| PM | Male | 7 | 2 | Dry | 3 | 3 | 4 | 5 | 4 | 5 | 4 | 4±0.8 |
| PM | Male | 7 | 2 | Dry | 3 | 3 | 4 | 5 | 4 | 4 | 4 | 3.9±0.7 |
| PM | Male | 7 | 2 | Wet | - | 3 | 4 | 4 | 4 | 4 | 4 | 3.8±0.4 |
| TH | Male | 6 | 2 | Dry | 2 | 2 | 3 | 3 | 3 | 3 | - | 2.7±0.5 |
| PM | Male | 7 | 3 | Dry | 3 | 3 | 4 | 5 | 4 | 4 | 4 | 3.9±0.7 |
| PM | Male | 7 | 3 | Wet | 4 | 4 | 5 | 5 | 4 | 5 | 5 | 4.6±0.5 |
| PM | Male | 7 | 3 | Wet | 4 | 4 | 5 | 5 | 4 | 5 | 5 | 4.6±0.5 |
| TH | Male | 6 | 3 | Wet | 4 | 4 | 5 | 5 | 4 | - | - | 4.4±0.5 |
| TA | Female | 6 | 1 | Dry | 1 | 2 | 3 | - | 3 | - | - | 2.3±0.9 |
| TA | Female | 6 | 1 | Wet | 2 | 2 | 3 | 3 | 3 | 3 | 3 | 2.7±0.5 |
| TA | Female | 6 | 1 | Wet | 2 | 2 | 3 | 3 | 3 | 3 | 3 | 2.7±0.5 |
| TA | Female | 6 | 1 | Wet | 2 | 2 | 3 | 3 | 3 | 3 | - | 2.7±0.5 |
| TA | Female | 6 | 1 | Dry | 2 | 3 | 3 | 3 | 3 | 3 | - | 2.8±0.4 |
| PR | Female | 5 | 1 | Wet | 1 | 1 | 2 | 3 | 3 | 3 | 2 | 2.1±0.9 |
| PR | Female | 5 | 1 | Wet | 1 | 1 | 2 | 3 | 3 | 3 | 2 | 2.1±0.9 |
| PR | Female | 5 | 1 | Wet | 1 | 2 | 2 | 3 | 3 | 3 | 2 | 2.3±0.8 |
| PR | Female | 5 | 1 | Wet | 1 | 2 | 2 | 3 | 3 | 3 | 2 | 2.3±0.8 |
| PR | Female | 5 | 1 | Dry | 1 | 3 | 3 | 3 | 3 | 3 | - | 2.7±0.8 |
| WA | Female | 7 | 1 | Wet | 1 | 1 | - | - | 2 | 2 | 2 | 1.6±0.5 |
| WA | Female | 7 | 1 | Wet | 2 | 2 | 2 | 2 | 3 | 3 | 3 | 2.4±0.5 |
| WA | Female | 7 | 1 | Dry | 3 | 3 | 3 | 3 | 4 | 3 | - | 3.2±0.4 |
| TA | Female | 6 | 2 | Dry | 2 | 3 | 3 | 3 | 3 | 3 | - | 2.8±0.4 |
| TA | Female | 6 | 2 | Wet | 2 | 3 | 4 | 4 | 3 | 3 | - | 3.2±0.8 |
| WA | Female | 7 | 2 | Dry | 3 | 3 | 3 | 3 | 4 | 4 | 4 | 3.4±0.5 |
| TA | Female | 6 | 3 | Dry | 2 | 3 | 4 | 4 | 3 | 3 | 3 | 3.1±0.7 |
| TA | Female | 6 | 3 | Wet | 2 | 3 | 4 | 4 | 3 | 4 | 3 | 3.3±0.8 |
